# The Role of Alternative Splicing Factors hnRNP G and Fox-2 in the Progression and Prognosis of Esophageal Cancer

**DOI:** 10.1155/2022/3043737

**Published:** 2022-11-23

**Authors:** Yuanyuan Zheng, Xiaoyu Niu, Wenhua Xue, Lifeng Li, Qishun Geng, Zhirui Fan, Jie Zhao

**Affiliations:** ^1^Department of Pharmacy, The First Affiliated Hospital of Zhengzhou University, Zhengzhou, Henan 450052, China; ^2^Internet Medical and System Applications of National Engineering Laboratory, The First Affiliated Hospital of Zhengzhou University, Zhengzhou, Henan 450052, China; ^3^Department of Anesthesiology, Affiliated Cancer Hospital of Zhengzhou University, Zhengzhou, Henan 450052, China; ^4^Department of Oncology, The First Affiliated Hospital of Zhengzhou University, Zhengzhou, Henan 450052, China; ^5^Integrated Traditional and Western Medicine, The First Affiliated Hospital of Zhengzhou University, Zhengzhou, Henan 450052, China

## Abstract

**Aim:**

Alternative splicing (AS) has been widely demonstrated in the occurrence and progression of many cancers. Nevertheless, the involvement of cancer-associated splicing factors in the development of esophageal carcinoma (ESCA) remains to be explored.

**Method:**

RNA-Seq data and the corresponding clinical information of the ESCA cohort were downloaded from The Cancer Genome Atlas database. Bioinformatics methods were used to further analyzed the differently expressed AS (DEAS) events and their splicing network. Kaplan–Meier, Cox regression, and unsupervised cluster analyses were used to assess the association between AS events and clinical characteristics of ESCA patients. The splicing factors screened out were verified in vitro at the cellular level.

**Results:**

A total of 50,342 AS events were identified, of which 3,988 were DEAS events and 46 of these were associated with overall survival (OS) of ESCA patients, with a 5-year OS rate of 0.941. By constructing a network of AS events with survival-related splicing factors, the AS factors related to prognosis can be further identified. In vitro experiments and database analysis confirmed that the high expression of hnRNP G in ESCA is related to the high invasion ability of ESCA cells and the poor prognosis of ESCA patients. In contrast, the low expression of fox-2 in esophageal cancer is related to a better prognosis.

**Conclusion:**

ESCA-associated AS factors hnRNP G and Fox-2 are of great value in deciphering the underlying mechanisms of AS in ESCA and providing clues for therapeutic goals for further validation.

## 1. Introduction

According to the 2020 global cancer epidemiology report, esophageal cancer (ESCA) has the 7^th^ highest incidence. It is the 6^th^ leading cause of mortality, while ESCA mortality in developing countries rises [1]. Although the efficiency of ESCA's advanced diagnosis and multidisciplinary therapy has demonstrated a notable improvement recently, data from the last decade showed that the 5-year overall survival (OS) rates in the US, China, and Europe are 22.0%, 20.9%, and 12.6%, respectively. 70% of patients have missed the opportunity of undergoing radical surgery on the first diagnosis because of their late disease stage [2–5]. Related studies have shown that immunotherapy significantly prolongs the survival of advanced gastric or gastroesophageal junction cancer patients (8.2 months: 7.1 months, hazard ratio (HR) = 0.78, 95% confidence interval (CI: 0.63–0.96, *P* = 0.0095), but in all patients with ESCA, the effective rate is only 13.1% (41/314) [6]. Similarly, a phase III trial of epidermal growth factor receptor (EGFR) inhibitors has revealed no difference in OS between the two treatment groups (median gefitinib 3.73 months, 95% CI 3.23-4.50, placebo group 3.67 months, 95% CI 2.97-4.37; hazard ratio [HR] 0.90, 95% CI 0.74-1.09, *P* = 0.29) [7]. It is worth noting that accurate medicines and new biomarkers relying on genomic data provide new methods for the prevention, diagnosis, and therapy, which is an advancing area of research.

In recent years, the emergence of comprehensive studies on the genomics of ESCA, including whole-exome sequencing, gene mutation analysis, DNA methylation profiling, and deregulated pathways, has contributed to a deeper understanding of ESCA [8]. Developments in diversified clinical databases and high-throughput genomic technologies have made exploring cancer pathogenesis at the molecular level easier. Based on this situation, we explored the possible influencing factors of ESCA at the RNA level to identify more effective therapeutic targets, which are valuable to predict treatment response and prognosis.

Many precursor mRNAs are processed to produce only one mature mRNA, which is translated into a corresponding polypeptide, and some can be spliced into mRNAs with different structures. This phenomenon is called alternative splicing (AS). AS increases the use of a limited number of genes and is one of the mechanisms for increasing the diversity of biological proteins in multicellular eukaryotes [9]. In the extensive process from yeast meiosis to Drosophila circadian rhythm and mammalian neuronal differentiation, alternative splicing has shown the importance of regulating the expression of genes of different subtypes during growth and differentiation [10, 11]. AS plays a crucial role in biological processes, molecular functions, signal pathways, and cellular components. The disruption of AS likely results in abnormal cell differentiation even in cancer and other diseases [12]. AS occurs differently in different tissues; approximately 30% of AS is tissue-specific because the exon of genes is expressed difference tissues, and changes in AS will occur with the adaption of cancer progression [13, 14] Increasing evidence has shown that widespread splicing disorders result in the mutation of targeted genes and promote the progression of tumors [15, 16]. Therefore, a study of AS will identify potential biomarkers for cancer therapy.

## 2. Materials and Methods

### 2.1. ESCA Cell Line Culture

Human esophageal epithelial cell lines Het-1A and the ESCA cell lines (TE-1, KYSE-150, and EC-109) were all purchased from Shanghai cell Resource Center Academy of Sciences. Cells were cultured in a complete medium containing 90% DMEM (4 mM glutamine and 1% penicillin-streptomycin) and 10% fetal bovine serum at 37°C in a CO_2_ cell culture incubator. Cells were passaged at intervals of 2 to 3 days, and logarithmic growth phase cells were selected for the experiment.

### 2.2. RNA Isolation, Reverse Transcription, and Quantitative RT-PCR

Total RNA was extracted using TRIzol Reagent (American life Technologies) and detected the absorbance of RNA at 260 nm-280 nm with a spectrophotometer to obtain concentration value. Then, the total RNA extracted was reverse-transcribed into first-strand cDNA using a reverse transcription kit from Takara Corporation of Japan. Shanghai Biological Engineering Co., Ltd. synthesized the PCR primer, and more details can be found in Table 1. Using GAPDH mRNA as an internal reference and the 2 − *ΔΔ*CT method was employed to calculate the relative expression levels of the molecules. Reaction conditions were as follows: 95°C predenatured for 30 s, 95°C for 5 s, 60°C for 34 s, 95°C for 15 s, and 60°C for 60 s, a total of 40 cycles.

### 2.3. siRNA Transfection

EC-109 and KYSE-150 cells plated in 6-well plates (invasion assay) and 35 mm petri dish (transfection efficiency measurement) were transfected with different sequences of siRNAs (Table 2) targeting hnRNP G, FOX2, and IAH1 using reagent when 60% confluent. Add 0.6 *μ*g of plasmid and 4 *μ*l jetPRIME reagent from Polyplus (850 bd Sébastien Brant-67400 Illkirch-France) to 200 *μ*l jetPRIME buffer, spin down and incubate at room temperature for 10 minutes, and then add to 6-well plates (invasion assay) and 35 mm petri dish. The total RNA of the cells in the 35 mm petri dish was collected to verify the transfection results 48 hours after transfection, and the cells in 6-well plates were subjected to a wound healing test 24 hours after transfection.

### 2.4. Generation of Overexpressing Cell Lines

For LV IAH1/hnRNP G/FOX2-GFP-KYSE-150/EC109 cells, the target sequences were cloned into vectors PCDH-CMV-MCS-EF1-copGFP-T3A-Puro (Tsingke Biotechnology Co., Ltd.) and pLV-C-GFPSpark® (SinoBiological, HG25922-ACGLN) and transfected into 293 T cells. The medium was changed the following day, and the viral containing supernatant was collected 48 h after transfection, filtered through a 0.45 *μ*m filter (Millipore, SLHV033RB) and subsequently used to infect cells with polybrene (8 *μ*g/ml; Sigma, TR-1003-G). KYSE-150 and EC109 cells were infected by incubation with lentivirus-containing supernatant for 48 h. Transduced cells were purified by puromycin (Gibco, A1113803) selection. The transfection effect was observed under a fluorescence microscope. qPCR was performed to analyze the efficiency of IAH1/hnRNP G/FOX2 overexpression.

### 2.5. Cell Migration Assay

The transfected cells (5 × 10^5^ per well) were seeded onto six-well plates and incubated at 37°C in a 5% CO_2_ humidified incubator for 24 hours. Use a sterile tip pipette tip to make a smooth scratch on the cells perpendicular to the well plate, wash the exfoliated cell debris and take a picture under an inverted microscope, and record it as the result of 0 hours. Place the cells in a serum-free DMEM medium. The same marked field of view was photographed under the microscope at 24 and 48 hours. According to the cell scratch healing area, calculate the scratch healing rate of each group of cells.

### 2.6. AS Events from The Cancer Genome Atlas RNA Sequences

RNA sequence expression data are available at The Cancer Genome Atlas (TCGA) database. We obtained data on the AS events related to ESCA. We analyzed it using SpliceSeq, a java program that provides a comprehensive view of AS patterns and highlights their biological consequences [17]. AS events are sorted into seven patterns: exon skip (ES), mutually exclusive exons (ME), retained intron (RI), alternate promoter (AP), alternate terminator (AT), alternate donor site (AD), and alternate acceptor site (AA). The percent splicing in (PSI) value, ranging from 0 to 1, summarizes the rate of splicing a specific exon into the transcript population of a gene. The score of PSI indicated the AS events for specific exons without the need to know the comprehensive synthetic of full-length transcripts [18].

### 2.7. Analysis of Differential Variable Splicing Events

On analysis of the AS spectrum from ESCA and adjacent tissue samples, the AS events were required to fulfill the condition that a *t*-test yielded a *P* value of <0.05 and fold change (FC) > 1.5 or FC < 2/3.

### 2.8. Survival Analysis

Download the data of patients with complete clinical data and OS greater than 30 days from the TCGA database. To explore the effect of AS on the OS in ESCA patients, we divided the patients into two groups based on the median PSI. In order to remove any genes that may not be independent predictors of prognosis, LASSO Cox regression was used for further analysis of variable shear events associated with survival in seven types. ClusterProfiler analysis was used to conduct Gene Ontology (GO) enrichment analysis to identify the effects of survival-related AS events on biological processes, cellular components, and molecular functions. Multivariable Cox regression was conducted to analyze the difference between the seven patterns of AS events and OS. It then eliminated the genes that had no relevance with survival and confirmed the prognostic predictor. In addition, we plotted the accuracy of the receiver-operating characteristic (ROC) curves to compare the predictive models for each type of AS.

The online database UALCAN (http://ualcan.path.uab.edu/index.html), as a newly developed interactive web server, enables gene expression data of 184 ESCA from the TCGA database to be analyzed through standard processing [19].

### 2.9. Splicing-Related Network Construction

Through database filtering, a list of 67 human splicing factors was created [20]. The splicing factor gene expression in the mRNA splicing pathway was derived from grade 3 mRNA-seq data in TCGA. Survival analysis was performed to identify survival-related shear factors. The correlation between survival-related splicing factor gene expression and survival-related AS PSI values was analyzed using the Spearman test. *P* < 0.05 was defined as significantly correlated. The final interaction network of variable shear events and shear factors was constructed using Cytoscape (3.6.0).

### 2.10. Identifying the Effects of AS on the Prognosis of Patients with Different ESCA Subtypes

The unsupervised consensus method analyzed the association between ESCA subtypes and OS based on Consensus Cluster Plus.

## 3. Results

### 3.1. AS Events in ESCA

**Figure 1:**
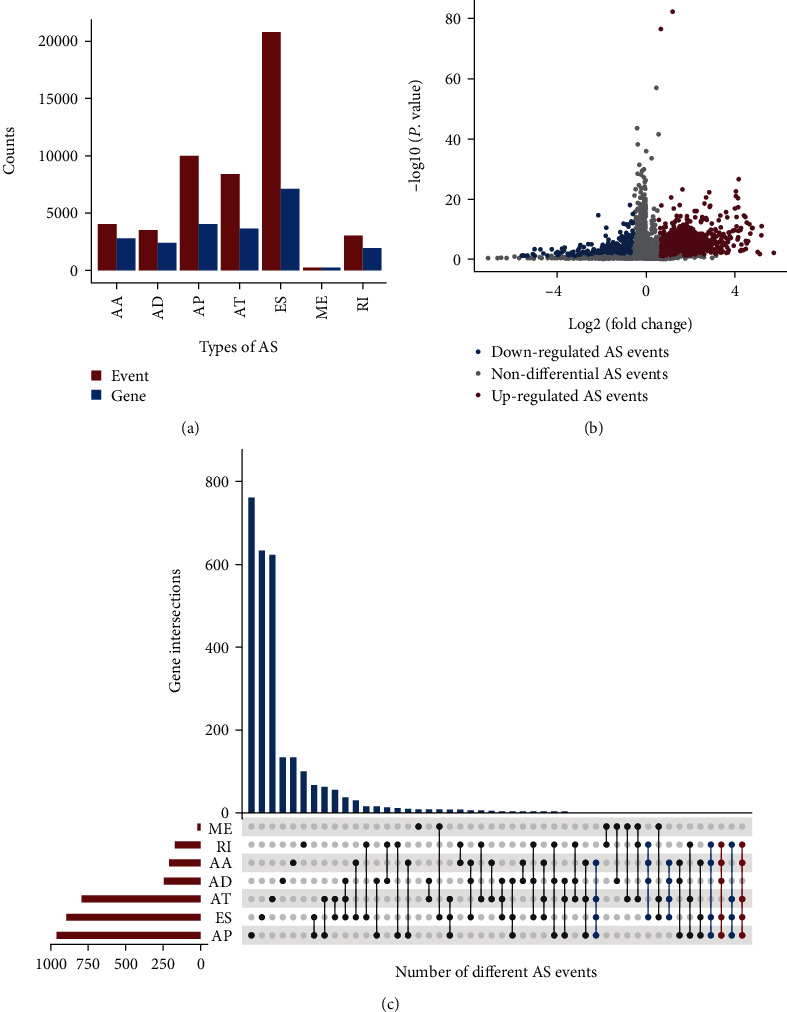
Differently expressed AS events in ESCA. (a) Each AS events type and its number of genes involved in ESCA. (b) Draw a volcano graph of differential variable splicing events. The red nodes represent the upregulated AS events, and the blue nodes represent the downregulated AS events; (c) The UpSet intersection diagram shows seven types of survival associated DEAS events.

Overall, 50342 AS events of 10765 genes were identified in patients with ESCA: 4144 AA events in 2870 genes, 2589 AD events in 2463 genes, 10033 AP events in 4046 genes, 8848 AT events in 3690 genes, 20842 ES events in 7173 genes, 245 MT events in 237 genes, and 3038 RI events in 2001 genes (Figure 1). These results indicated that a gene may have several types of mRNA splicing events, and a gene may have up to four types of variable splicing. ES was the highest in number, accounting for one-third of the AS events, followed by AP, AT, AA, AD, RI, and ME in succession (Figure 1(a)).

### 3.2. Differently Expressed AS in ESCA

There were 3988 differently expressed AS (DEAS) events in 2818 genes of patients with ESCA (2758 upregulated AS events and 1230 downregulated AS events, Figure 1(b)). Interestingly, the number of AS events and involved genes was nonmatched, implying that one gene underwent more than one type of splicing event. Some genes had up to four variable types on DEAS. The UpSet plot (Figure 1(c)) was used to visualize the overlapping different AS events among genes. In the differential expression analysis, 5269 differentially expressed genes were identified in ESCA (2711 upregulated differentially expressed genes and 2558 downregulated differentially expressed genes).

### 3.3. Survival-Associated Differential Genes in ESCA

**Figure 2:**
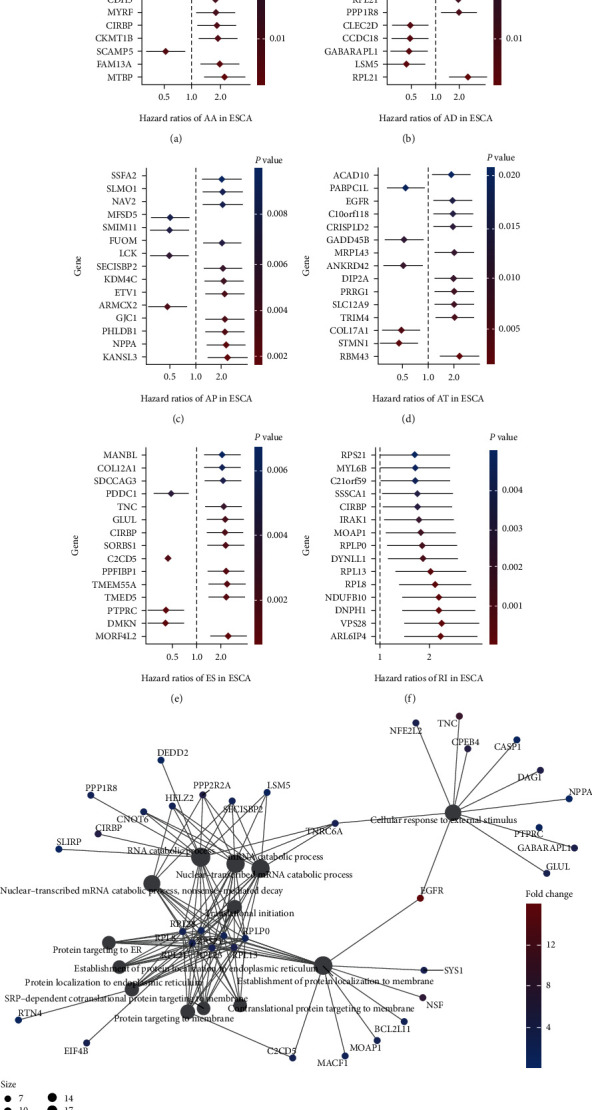
Differential spliced genes associated with ESCA survival in each AS type and their functional enrichment analysis results. (a)–(f) Survival-related top 15 AS events forest map in each AS type. (g) Functional enrichment analysis results of differentially spliced genes in ESCA from GO analysis.

To illustrate the relationship between the OS in patients with ESCA-related AS events, we assessed the predictive value of AS events in patients with ESCA using univariate Cox regression analysis in patients with intact clinical survival information. We detected a total of 217 survival-related AS events (*P* < 0.05) among all AS events that occurred in ESCA. A forest map of the differential gene expression in the six types of AS events was plotted based on the top 15 survival-related AS events (Figures 2(a)–2(f)). The AS events included AA, AD, AP, AT, ES, and RI, except ME, in which there was only one splicing event associated with survival (ZFP2, HR = 0.56, 95% CI: 0.35–0.91). Most of these survival-related AS events were adverse prognostic factors. For instance, RI events showed a negative association with the survival of patients with ESCA (HR > 1). The forest maps revealed the genes participating in ESCA carcinogenesis. The EIF4B in AA of splicing events predicted poor survival of patients with ESCA. Other studies have experimentally validated that the EIF4B results in poor prognosis of tumor patients, increasing the credibility of our data [21, 22]. RPS21 and MYL6B in RI were associated with poor prognosis of patients with ESCA; they have been validated as oncoproteins in prostate tumor and hepatocellular carcinoma, respectively [23, 24]. We conducted GO functional enrichment analysis on 206 genes involved in AS events in ESCA further to explore the underlying molecular mechanism among the survival-associated events. The GO categories showed that the genes in survival-associated AS events were significantly enriched in different biological processes, such as translational initiation, mRNA catabolic processes, and cellular responses to external stimuli, among others (Figure 2(g)).

### 3.4. Prognostic Predictors for Patients with ESCA

**Figure 3:**
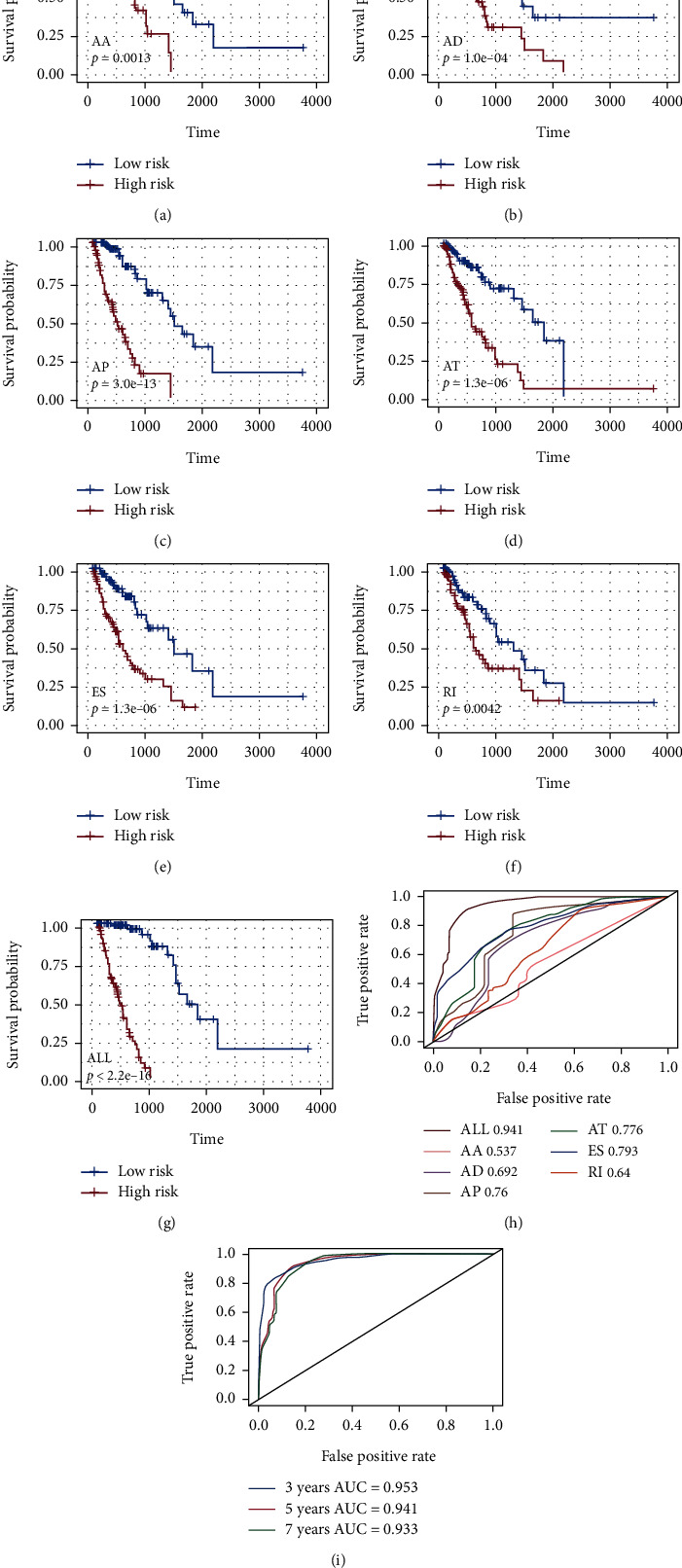
Relationship between AS events and prognosis of esophageal cancer and its predictive effect. (a)–(f) Kaplan–Meier curves of prognostic models built with AS events of AA, AD, AP, AT, ES, and RI splice types for patients with ESCA. The blue line indicates the low-risk group, whereas the red line indicates the high-risk group. (g) Kaplan–Meier curves of prognostic models built with all types of AS events. (h) The ROC curves of predictive models for each splicing type. (i) The ROC curves of predictive models for all splicing types over 3 years, 5 years, and 7 years of survival.

Considering the interaction between AS events and the prognosis of patients with ESCA, we attempted to verify if the six types of AS events could serve as the predictive element of ESCA by choosing the highly correlated AS events as the candidates to analyze the predictive factors in ESCA. AA and RI each have one prognostic factor, AD has three prognostic factors, AP has 22 prognostic factors, the independent prognostic factors were associated with the AT 6, and ES has 13 prognostic factors and did not identify the independent prognostic factors related to ME. According to our data, each splicing type performed well in predicting the prognosis of patients with ESCA (all adjusted *P* < 0.05, Figures 3(a)–3(f)). The ROC was applied in the prognostic models to confirm better the validity of AS events in forecasting the prognosis of patients with ESCA. In ESCA, the AS events, including AA, AD, AP, AT, ES, and RI, all demonstrated areas under the curve (AUCs) of the ROC curves of greater than 0.6, indicating powerful efficiency in distinguishing the prognosis of patients. ES showed the best performance in predicting the survival of ESCA patients (Figure 3(h)). In addition, variable shear events associated with survival were derived to construct final prognostic predictors. Notably, the final prognostic model showed the best performance in predicting outcomes (*P* < 0.05, ROC = 0.843), as shown in Figures 3(h) and 3(g) (Supplemental materials. 1). Meanwhile, we compared ROC curves of 3, 5, and 7 years of the final prognosis model, and it can be seen that the model has good predictive ability (Figure 3(i)).

### 3.5. Network of Survival-Associated AS Events and Splicing Factors

**Figure 4:**
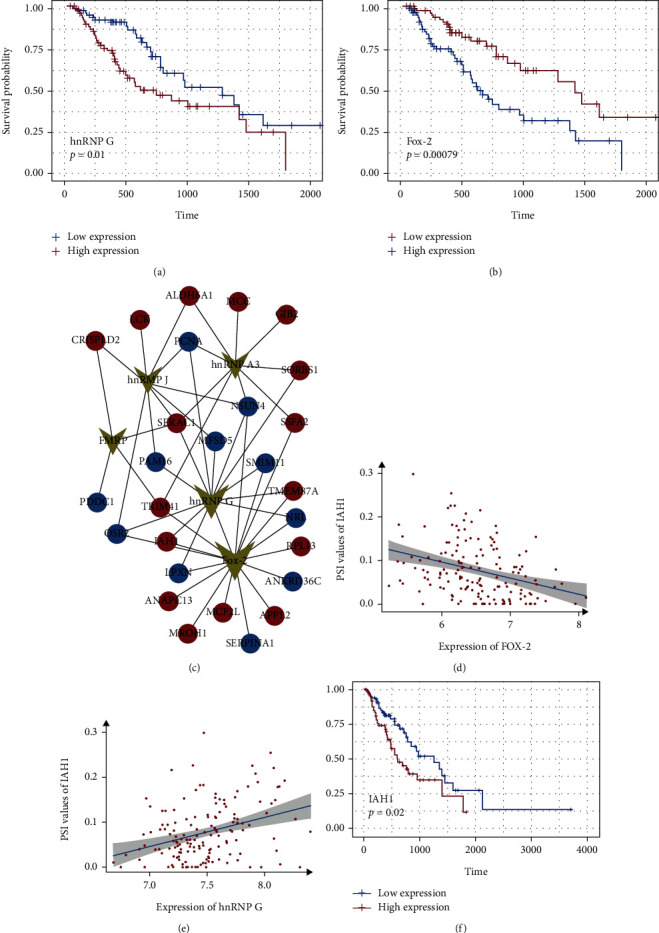
Network of survival-associated AS events and splicing factors. (a, b) Kaplan-Meier curves for splicing factor hnRNP G and Fox-2 with high (red) and low (blue) expression groups in patients with ESCA, respectively. (c) Splicing correlation network constructed by Cytoscape. Five survival-associated splicing factors (green dots) in AS events were upregulated (red dots) or downregulated (blue dots) by involved genes. (d) Negative correlations between Fox-2 expression and the AP PSI value of IAH1. (e) Positive correlations between hnRNP G expression and the AP PSI value of IAH1. (f) Kaplan-Meier curves for splicing gene IAH1 with high (red) and low (blue) expression groups in ESCA patients.

To determine the specific splicing factors in the AS events associated with the survival of patients with ESCA, we conducted survival analysis based on gene expression of the splicing factors. Five splicing factors (hnRNP J, hnRNP A3, hnRNP G, FMRP, and Fox-2) showed a significant correlation with OS in patients with ESCA. The representative splicing factors and survival-associated AS events are shown in Figure 4(c). The Spearman test was also used to study the correlation between the PSI of AS events and splicing factors associated with survival. The correlation network indicated that a total of 27 survival-associated AS events were highly correlated with five splicing factors (green points), with 16 positive (red points), and 11 negative (green points) AS events (Figure 4(c)). Splicing factors such as Fox-2 and hnRNP G were involved in 17 and 13 AS events, respectively. Among these survival-associated splicing factors, some were associated with poor prognosis, whereas others were linked with favorable prognosis (Figures 4(a) and 4(b)). AS events (IAH1, NSUN4, SERAC1, and TRIM4) were correlated with up to four splicing factors. Among them, IAH1 was a gene that is related to hnRNP G (positive correlation, Figure 4(d)) and Fox-2 (negative correlation, Figure 4(e)), and its high expression can cause poor prognosis of patients with ESCA (Figure 4(f)). Downregulation of the hnRNP G expression and upregulation of the FOX2 expression in esophageal cancer cells were found by silencing IAH1 (Supplementary Figure 1A-1C). Furthermore, knockdown of hnRNP G resulted in upregulation of IAH1, while knockdown of FOX2 resulted in downregulation of IAH1 (Supplementary Figure 1D, 1E). Stable cell lines overexpressing IAH1, hnRNP G, and FOX2 were constructed, respectively (Supplementary Figure 1F), and it was found that the overexpression of IAH1 resulted in upregulation of hnRNP G and downregulation of FOX2 (Supplementary Figure 1G). The overexpression of hnRNP G led to the upregulation of IAH1, while the overexpression of FOX2 led to the downregulation of IAH1 (Supplementary Figure 1H, 1I).

### 3.6. Expression of Splicing Factors Related to Prognosis of ESCA Cell Lines Is Involved in ESCA Progression and Affects Its Prognosis

**Figure 5:**
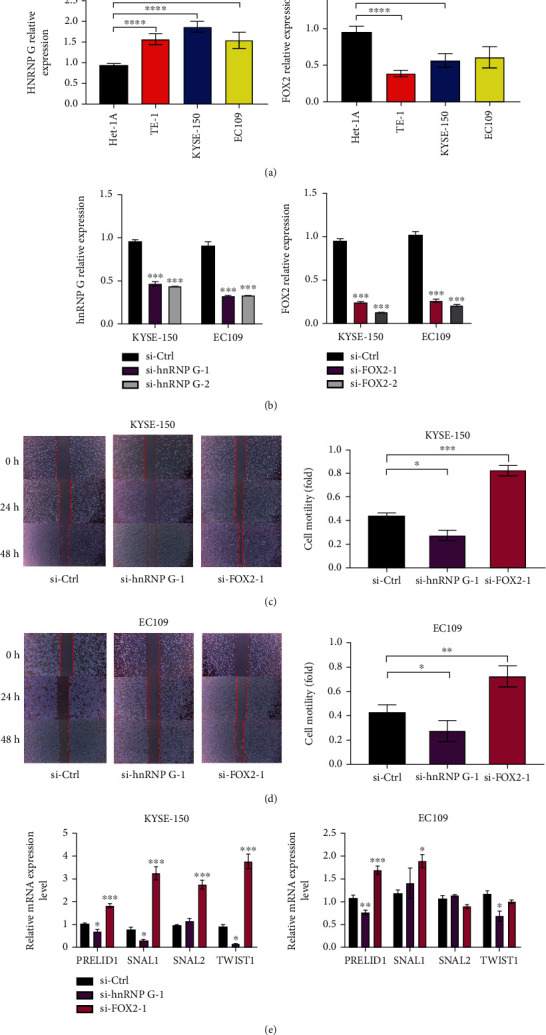
The effect of hnRNP G and FOX2 on the biological behavior of ESCA cell lines in vitro. (a) The expression of hnRNP G and FOX2 in ESCA cell lines (TE-1, KYSE-150, EC-109) and normal esophageal epithelial cell line HET-1A was detected by qRT-PCR. ^∗^*P* < 0.05, ^∗∗^*P* < 0.01, ^∗∗∗^*P* < 0.001, and ^∗∗∗∗^*P* < 0.0001. (b) The qRT-PCR analysis confirmed that the expression of hnRNP G and FOX2 in EC-109 and KYSE-150 cells was reduced compared with cells transfected with control siRNA sequences. (c, d) Invasion ability was measured in EC-109, and KYSE-150 transfected with Ctrl, FOX2, and hnRNP G sequences by wound healing test. ^∗^*P* < 0.05 vs. cells transfected with control siRNA. (e) The bar graphs represent the mRNA expression level of EMT-related genes in EC-109 and KYSE-150 cell lines as determined by real-time PCR. ^∗^*P* < 0.05, ^∗∗^*P* < 0.01, ^∗∗∗^*P* < 0.001. All data were representative of at least three independent experiments (*n* = 3; error bar, SD).

Next, we examined the expression levels of the splicing factors Fox-2 and hnRNP G in the ESCA cell lines and normal esophageal epithelial cells. qRT-PCR results showed that the expression of Fox-2 in ESCA cell lines KYSE-150, EC-109, and TE-1 was significantly lower than that of normal esophageal epithelial cell line HET-1A (Figure 5(a)). In comparison, the expression of hnRNP G in ESCA cell lines KYSE-150, EC-109, and TE-1 was significantly higher than that of normal esophageal epithelial cell line HET-1A (Figure 5(a)). The consistency between the results of RT-PCR and the above survival analysis demonstrated that our results were reliable. The siRNAs sequences were transfected into ESCA cell lines to establish Fox-2 and hnRNP G gene knockout models to analyze the effects of splicing factors Fox-2 and hnRNP G on tumor behavior. qPCR experiment detects the transfection effect (Figure 5(b)). The migration and invasion ability of si-hnRNP G cells was not as good as the control, and the difference has statistically significant (EC-109, *P* = 0.0312; KYSE0-150, *P* = 0.0135). The migration and invasion ability of si-FOX2 cells was higher than that of the control, and the difference was statistically significant (^∗^*P* < 0.05, ^∗∗^*P* < 0.01, ^∗∗∗^*P* < 0.001) (Figures 5(c) and 5(d)). Then, by verifying the mRNA levels of epithelial-mesenchymal transition- (EMT-) related genes, we found that the expression levels of EMT-related genes were downregulated in si-hnRNP G cells. At the same time, they were upregulated in si-FOX2 cells (Figure 5(e)), which was consistent with the consequence of the previous wound-healing experiment. The same experiment was performed on another sequence of siRNA, and the results of the wound healing assay (Supplementary Figure 2A, 2B) and the expression level of EMT-related RNA (Supplementary Figure 2C) were consistent with those of the other sequence.

**Figure 6:**
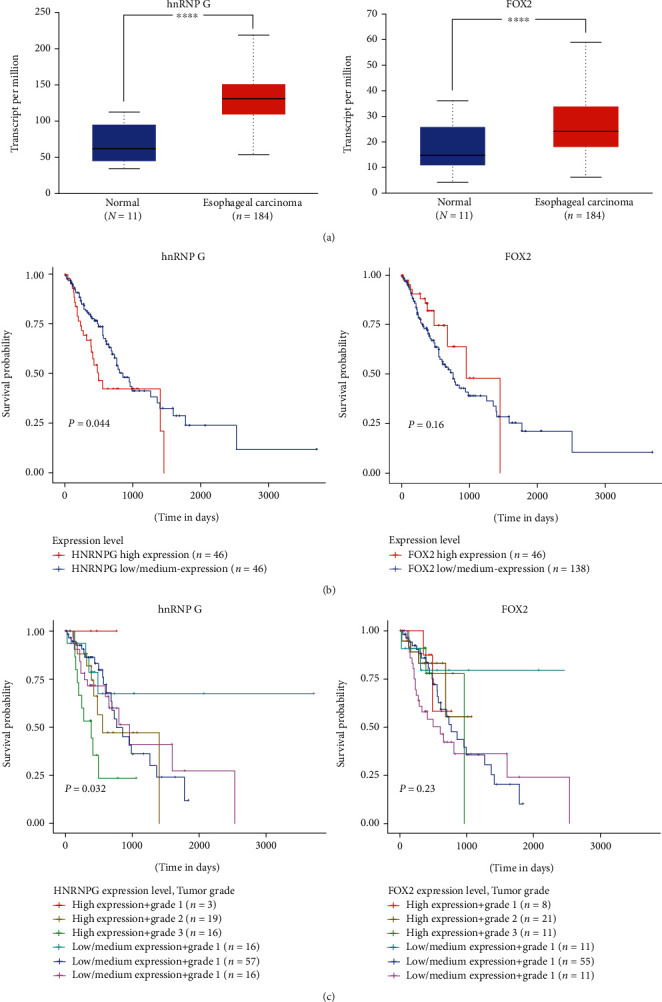
Effects of hnRNP G and Fox-2 expression on overall survival in ESCA. (a) Expression of hnRNP G (left panels) and FOX2 (right panels) in ESCA based on sample types. (b) Effect of hnRNP G (left panels) and FOX2 (right panels) expression level on ESCA patient survival. (c) Result of hnRNP G (left panels) and FOX2 (right panels) expression level and tumor grade on ESCA patient survival.

The analysis of ESCA patients in the TCGA database found that the splicing factors both hnRNP G and Fox-2 are highly expressed in ESCA patients (Figure 6(a)). Still, the splicing factor hnRNP G was negatively correlated with the OS of ESCA patients. The difference is statistically significant (*P* = 0.044), while the splicing factor Fox-2 was positively correlated with the OS of ESCA patients, and the difference was not statistically significant (*P* = 0.16) (Figure 6(b)). Through further analysis of different grades of esophageal cancer, it was found that hnRNP G concentrated in patients with grade 2 ESCA. Regardless of the high expression group or the low expression group, the OS was significantly shorter than early ESCA patients (*P* = 0.032) (Figure 6(c)). There were more patients with low Fox-2 expression than those with high expression. Among patients with ESCA of the same stage, patients with low expression of FOX-2 have a longer survival time, and the difference was not statistically significant (*P* = 0.23) (Figure 6(c)).

### 3.7. Molecular Subtype Clustering

**Figure 7:**
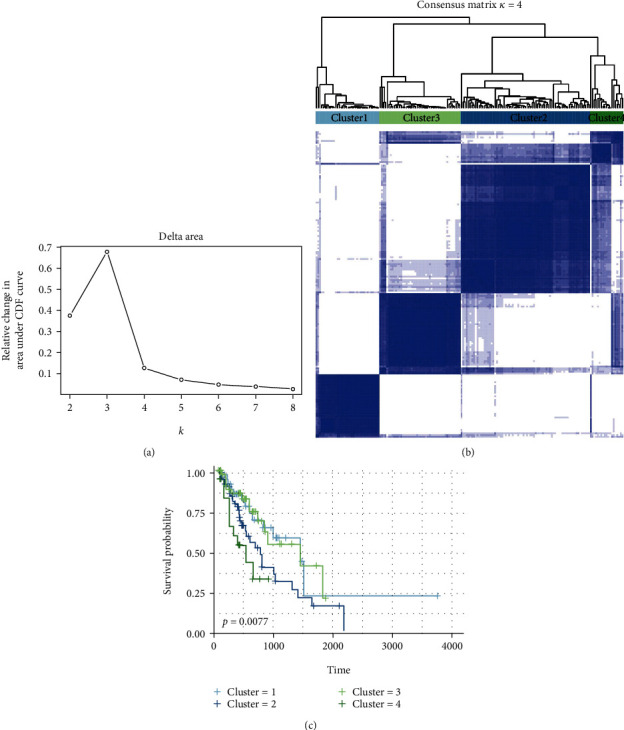
Molecular subtype clusters associated with prognosis obtained by AS events. (a) Statistical analysis of elbows for different numbers of clusters (*k* = 2 to 8). (b) The consensus matrix heat maps and consensus values ranging from 0 (white, no samples aggregation) to 1 (blue, sample always aggregation). (c) Survival analysis in the identified four clusters.

The occurrence of variable splicing events differs greatly at the individual level. To obtain a robust classification, we use the unsupervised consensus method implemented by Consensus Cluster Plus to identify the molecular subtypes of esophageal cancer. We identified different AS types according to 46 survival-related AS events by performing unsupervised analysis on all samples. Here, we introduced the functional heat map, which revealed the hidden trends driven by different AS types and reduced manual labor in discovering and comparing different patterns [25]. Using the Elbow method to determine the optimal number of clusters, and based on the distribution of the consensus values ranging from 0 (white, no samples aggregation) to 1 (blue, sample always aggregation), we finally determined four sets of samples: C1 (*n* = 36, 20.7%), C2 (*n* = 73, 42.0%), C3 (*n* = 46, 26.3%), and C4 (*n* =19, 11.0%) (Figures 7(a) and 7(b)). Kaplan–Meier analysis was then used to explore the correlation between these clusters and the survival rate of patients with ESCA, indicating that the clusters were significantly associated with the outcome of survival (Figure 7(c)). Therefore, we can acquire molecular subtype clusters associated with prognosis through a small number of AS events.

## 4. Discussion

More than 95% of precursor messenger RNA (pre-mRNA) are processed to multiple mRNAs through AS events [26]. Growing evidence has demonstrated that AS plays a crucial role in physiological processes and cell development programs and the differentiation of cells, leading to the development of tumors and other diseases [12, 27]. AS events related to abnormal pre-mRNA precursors have been widely demonstrated in tumor occurrence, increased aggressiveness, drug resistance, and other aspects of tumor progression. These events include changes in splicing types and mutations in splicing factors and regulatory signals [28]. In the past several years, growing evidence has indicated that AS events promise to recapitulate cancer epigenetics [29, 30]. For example, the key regulator of AS events in lung cancer, RNA-binding protein QKI, is significantly associated with poor prognosis when downregulated [31].

Similarly, changes in AS events in lung cancer will also affect the transcripts of VEGFA, MACF1, APP, and NUMB genes, thereby promoting the process of tumorigenesis [32]. Mutations of SF3B1-encoding proteins involved in RNA splicing may be a driving factor and novel therapeutic target in breast cancers [33]. Similarly, breast tumors adopt AS events to remove deleterious germline BRCA1 mutations by removing exon 11 to contribute to retaining activity and drug resistance [34]. Therefore, mutations in splicing factors and regulatory pathways in AS events can lead to abnormal splicing types and different results, including tumor development, invasion, replacement, and transformation. Therefore, the primary functional study of AS events and mechanisms of tumorigenesis and development can facilitate the discovery of novel biomarkers.

In this study, we identified AS events and profiles of regulatory splicing factors and established the interrelation between them in ESCA through an analysis of the TCGA program. A total of 50342 AS events of 10765 genes were detected in samples of tumors with seven splicing types (AA, AD, AP, AT, ES, ME, and RI), of which ES was the most common splicing type. It seems that AS event is an ordinary process in ESCA, and that most splicing patterns are active. There are 3988 DEAS events between ESCA and nontumor tissues: 2758 upregulated AS events and 1230 downregulated AS events involving 2818 genes. Then, we found that 217 AS events among those DEAS events were significantly associated with the survival rate of patients with ESCA. Most of these AS events showed a critical influence on tumor biology. The top 15 splicing events of RI in DEAS events lead to the poor survival of patients with ESCA, which is consistent with some findings regarding AS events and tumor prognosis [35]. In addition, GO functional enrichment and KEGG pathway analysis provided insights into the enrichment of many DEAS events in many biological adhesions, cell organizations, and many other essential biological processes. Several genes (EIF4B, RPS21, MYL6B) have been demonstrated to make ESCA patients which have poor survival, and further investigation concerning their potential impact on ESCA is still required.

Moreover, we established a prognostic model for each splicing type using multivariate Cox regression analysis. Each type of AS event performed reasonably well in showing a positive or negative prognosis. The six survival-associated types (AA, AD, AP, AT, ES, and RI) of AS events showed different AUCs of ROC curves, and ES showed the maximum efficiency in predicting the survival of ESCA patients with the best AUC value: 0.793. The integrated predictive model with six types together showed a high correlation with survival. Further specific functional experiments to determine how alternative splicing modulates tumor prognosis are needed. Given that certain genes and splicing factors may exhibit extensive” spliceosome mutations,” leading to cancer-specific mis-splicing, we therefore focused on the correlation network of splicing factors and survival-associated AS events [36]. Five splicing factors (hnRNP A3, hnRNP J, hnRNP G, FMRP, Fox-2) with four AS events (IAH1, NSUN4, SERAC1, and TRIM4) showed a strong correlation with prognosis.

The immunohistochemistry staining of heterogeneous nuclear ribonucleoprotein G (hnRNP G) is more prominent in the normal oral cavity than in premalignant and malignant human oral tissue. hnRNP G exhibited tumor suppressor activity, including inhibition of cell proliferation, cell capacity, and enhancement of DNA repair capabilities in human oral squamous cell carcinoma (HOSCC) [37]. Another research showed a similar result that the hnRNP G protein nuclear expression was found higher in earlier endometrial cancer (EC) and patients without distant organs, and the high expression of hnRNP G in mRNA and protein levels indicated a favorable outcome for EC patients [38]. It is consistent with our findings that the expression of hnRNP G in ESCA cells is higher than that in normal esophageal epithelial cells, and several public datasets confirmed that the high expression of hnRNP G forebode poor prognosis for ESCA patients. The difference of exon sequence determined both the cell reactiveness and protein specificity. hnRNP G works in the regulation of Tra2-dependent splicing under the interaction of the splicing activator protein hTra2*β*, and differences in the radio of hnRNP G/Tra2*β* mRNA have been found in different tissues of the human body, which may indicate that hnRNP G possesses the cellular splicing preferences [39].

Castle et al. found that the AS events are tightly controlled in mammalian, while are exceptionally variable to genetic and environmental variability (such as the tumor), using the quantitative reverse transcription-PCR amplification [40]. Venables et al. used custom-built whole-transcript microarrays to establish a compendium of human AS events, including 24,426 AS events in 48 diverse human samples. The result showed the enrichment of Fox-2 across most tissues and cell lines [41]. Consistent with the consequence preceding, Das et al. conducted an immunohistochemical and qPCR analysis of breast and ovarian cancer tissues and found that AS events associated with cancer are driven by the expression level of Fox-2 [42]. Data analysis of human exon microarrays showed that no matter which forms of AS events, it will affect the expression of FOX-regulated ASE encoding, myosin, kinesin, and microtubule machinery and transporter-related proteins, which indicated the potential of FOX in regulating the plasticity and motility of cells and may be related to tumor metastasis and increased aggressiveness [43]. Consistent with the findings of other investigations, the splicing factors hnRNP A3 [44], FMRP [45], and Fox-2 [36] may be related to the development of tumors. The increased expression of NSUN4 and SERAC1 has been described in breast cancer [46]. The differential expression of TRIM4 makes cells sensitive to H_2_O_2_-induced death, which is common in tumor cell lines [47]. There is no evidence that IAH1 plays a key role in tumor development, but IAH1 (a homolog of isoamyl acetate hydrolytic esterase) has been shown to regulate the expression of genes involved in cholesterol synthesis, thereby affecting lipid metabolism [48]. According to our results, the IAH1 expression was positively correlated with the splicing factor hnRNP G and negatively correlated with the splicing factor FOX2 and was associated with poor prognosis. How IAH1 affects the progression of esophageal cancer may be an emerging target for future treatment of esophageal cancer.

To this end, we verified the expression of splicing factors related to prognosis in esophageal cells. The results showed that splicing factors hnRNP G negatively correlated with prognosis were highly expressed in ESCA cell lines. In contrast, the expression of splicing factors Fox-2 positively associated with prognosis in ESCA cell lines was significantly lower than that of normal esophageal epithelial cells. By transfecting siRNAs sequences to construct knockdown cell lines, Fox-2 deletion was shown to increase the invasiveness of ESCA cells. Correspondingly, this study also proved that the absence of hnRNP G can significantly reduce the invasion ability of ESCA cells; this trend can be seen from the images of the cell wounding healing test. In addition, TCGA database analysis showed that hnRNP G and FOX-2 were highly expressed in ESCA patients. In addition, all ESCA patients with high hnRNP G expression had a shorter median survival time, while patients with high FOX-2 expression had a longer median survival time. In the analysis of hnRNP G and tumor grade on ESCA patient survival, the higher expression of hnRNP G in the same grade has the worse survival. On the contrary, both the high and low Fox-2 expression groups were concentrated in grade 2 of ESCA, and the higher Fox-2 expression was, the better the prognosis was. In the future, the sample size can be expanded to confirm its impact on the overall survival and progression-free survival of ESCA patients, and the mechanism of action can be explored through basic experiments.

## 5. Conclusion

In summary, this was a comprehensive and up-to-date profile of AS events between ESCA and its corresponding nontumor tissues, uncovering the interevent correlations in splicing factors and prognostic signatures in ESCA. The interaction network of splicing factors and prophetic AS events highlights AS events' value and ESCA's tumorigenesis at the genome level. This analysis of survival-associated splicing factors and tumor-specific AS events also points out that new underlying clinical biomarkers still need to be validated in future mechanistic research and clinical trials. These splicing factors and genes can serve as potential prognostic biomarkers to guide the clinical treatment for ESCA patients and expound a novel etiology of ESCA in the future.

## Figures and Tables

**Table 1:** Primer sequences used for qRT-PCR.

FOX2	F: TACAGTGACGGTTATGGCAGG
	R: CCTCGGTATAAACTCGCCACA

hnRNP G	F: CCATCAAGAGGCTATGGCGAT
	R: CCCTCGTGTAAGTGGAGCA

IAH1	F: AGCCGTCAGACTGCTACAG
	R: AAAAGACTCGCCAAGTCATTGT

PRELID1	F: CAATGTTGCTCACTCGGTGTA
	R: GGTGAAGGTAGTCATGGTCTGA

SNAI1	F: TCGGAAGCCTAACTACAGCGA
	R: AGATGAGCATTGGCAGCGAG

SNAI2	F: CGAACTGGACACACATACAGTG
	R: CTGAGGATCTCTGGTTGTGGT

TWIST1	F: GGACAAGCTGAGCAAGATTCA
	R: CGGAGAAGGCGTAGCTGAG

GAPDH	F: GACCACAGTCCATGCCATCAC
	R: GTCCACCACCCTGTTGCTGTA

**Table 2:** Primer sequences for transfection.

si-FOX2-1	F: CCGGUGAGCAUAACCUGACACUCUA
	R: UAGAGUGUCAGGUUAUGCUCACCGG

si-FOX2-2	F: GCAAAUGGUUGGAAAUUAAGC
	R: UUAAUUUCCAACCAUUUGCAU

si-hnRNP G-1	F: GAUUUGUACCAUUCUUCUGTT
	R: CAGAAGAAUGGUACAAAUCCA

si-hnRNP G-2	F: CGUGAUGACUAUCCAUCAAGA
	R: UUGAUGGAUAGUCAUCACGUG

si-IAH1-1	F: CUGCGAACCUAAAGAGCAUTT
	R: AUGCUCUUUAGGUUCGCAGTT

si-IAH1-2	F: GCGAAGAACAGUGCAUCAUTT
	R: AUGAUGCACUGUUCUUCCCTT

## Data Availability

Previously reported public data were used to support this study and are available at https://portal.gdc.cancer.gov/ and http://ualcan.path.uab.edu/index.html. These prior studies (and datasets) are cited at relevant places within the text as references [17, 19].

## Supplementary Materials

Supplementary Materials Supplementary Figure 1: expression level of IAH1, hnRNPG, and FOX2 in ESCA. (a) The qRT-PCR analysis confirmed that the expression of IAH1 in EC-109 and KYSE-150 cells was reduced compared with cells transfected with control siRNA sequences. (b) The RNA expression of hnRNPG in si-IAH1-1 transfected cells. KYSE-150 cells were significantly downregulated compared with the control group, and EC109 cells were also downregulated, but there was no statistical difference. (c) The expression level of fox2 RNA was detected in siIAH1 transfected cells. KYSE150 cells and EC109 cells were significantly up-regulated. (d) IAH1 was significantly downregulated by si-hnRNPG transfection in KYSE150 cells and EC109 cells. (e) IAH1 was significantly upregulated by si-hnRNPG transfection in KYSE150 cells and EC109 cells. (f) Cell lines stably overexpressing IAH1, hnRNPG, and FOX2 were constructed, and the green fluorescence could be observed under the microscope after successful transfection of the plasmid. (g) The hnRNPG expression was upregulated, and the FOX2 expression was downregulated in IAH1 overexpressing cell lines. (h) Upregulation of IAH1 was found in cell lines overexpressing hnRNPG. (i) Downregulation of IAH1 was found in cell lines overexpressing FOX2. All data were representative of at least three independent experiments (*n* = 3; error bar, SD). Supplementary Figure 2: the effect of hnRNP G and FOX2 on the biological behavior of ESCA cell lines in vitro. (a) Invasion ability was measured in EC-109, and KYSE-150 transfected with another FOX2 and hnRNP G sequences by wound healing test. ^∗^*P* < 0.05 vs. cells transfected with control siRNA. (e) The bar graphs represent the mRNA expression level of EMT-related genes in EC-109 and KYSE-150 cell lines after transfected with another FOX2 and hnRNP G sequences. ^∗^*P* < 0.05, ^∗∗^*P* < 0.01, ^∗∗∗^*P* < 0.001. All data were representative of at least three independent experiments (*n* = 3; error bar, SD).
