# Supplementary figures and images for: The Role of Alternative Splicing Factors hnRNP G and Fox-2 in the Progression and Prognosis of Esophageal Cancer
Source: Dis Markers. 2022 Nov 23;2022:3043737. doi: 10.1155/2022/3043737 (PMC9712015; doi:10.1155/2022/3043737)

**Supplementary materials 1**


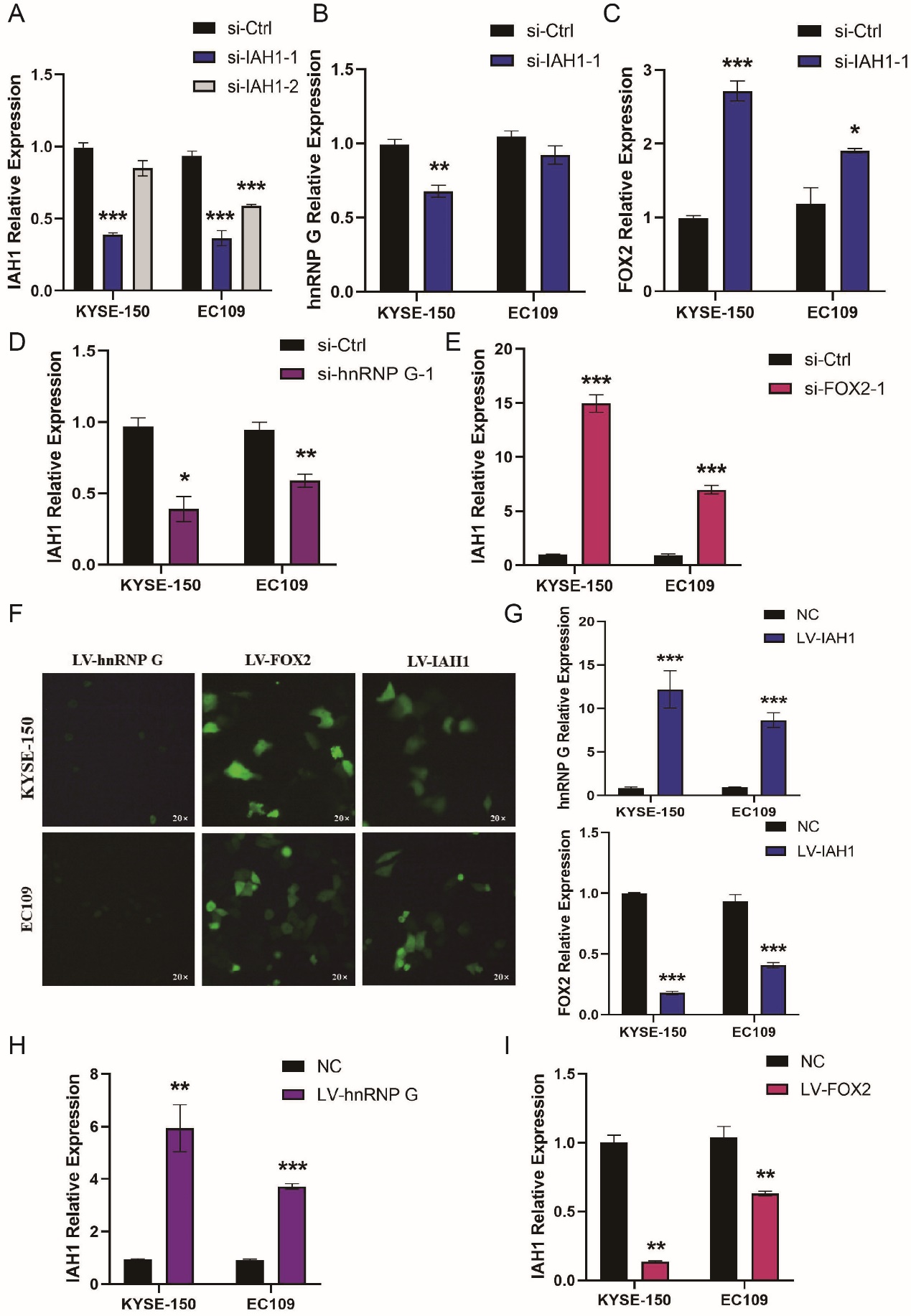


**Supplementary materials 2**


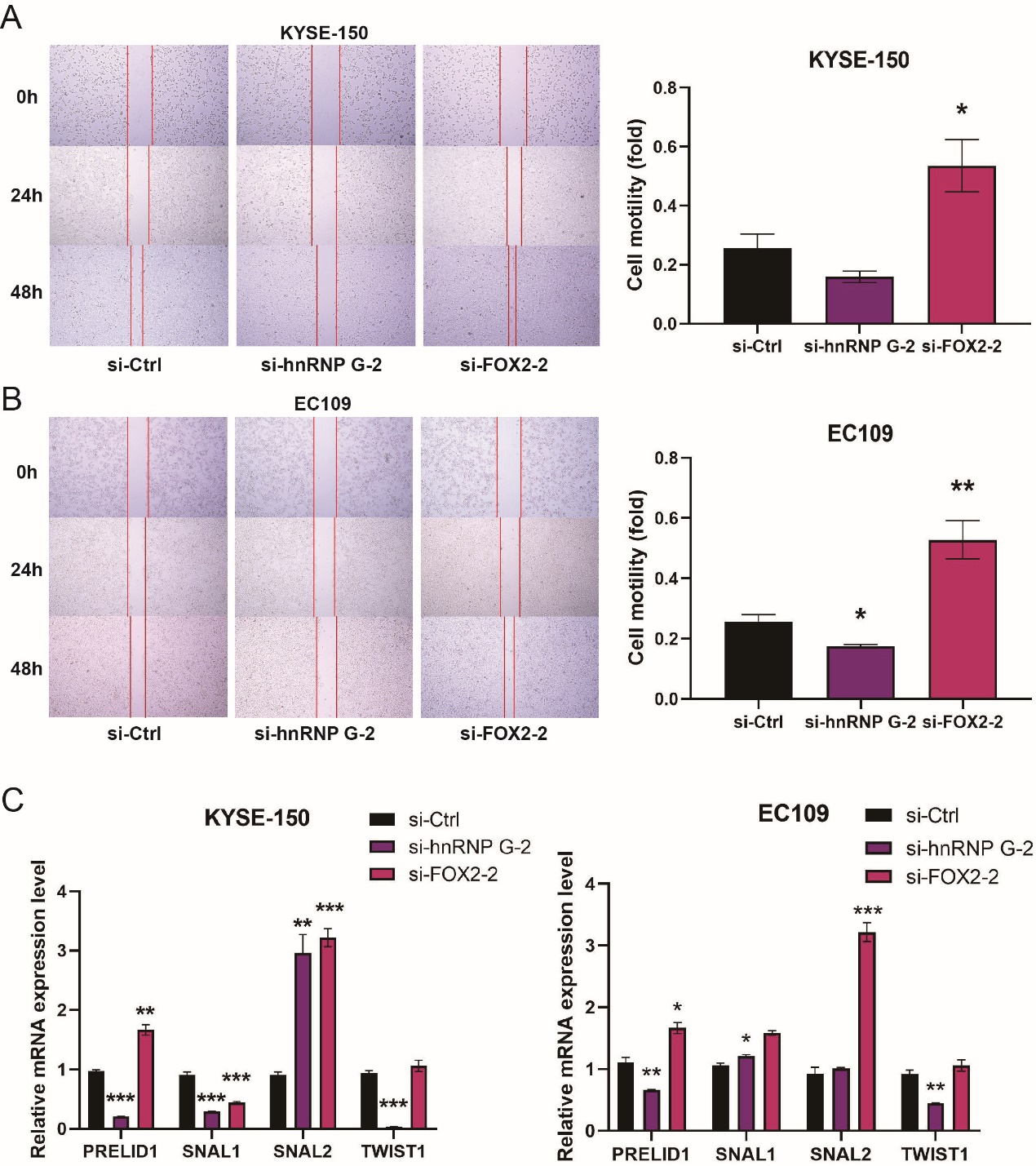

Supplement: Supplementary Materials — Supplementary Figure 1: expression level of IAH1, hnRNPG, and FOX2 in ESCA. (a) The qRT-PCR analysis confirmed that the expression of IAH1 in EC-109 and KYSE-150 cells was reduced compared with cells transfected with control siRNA sequences. (b) The RNA expression of hnRNPG in si-IAH1-1 transfected cells. KYSE-150 cells were significantly downregulated compared with the control group, and EC109 cells were also downregulated, but there was no statistical difference. (c) The expression level of fox2 RNA was detected in siIAH1 transfected cells. KYSE150 cells and EC109 cells were significantly up-regulated. (d) IAH1 was significantly downregulated by si-hnRNPG transfection in KYSE150 cells and EC109 cells. (e) IAH1 was significantly upregulated by si-hnRNPG transfection in KYSE150 cells and EC109 cells. (f) Cell lines stably overexpressing IAH1, hnRNPG, and FOX2 were constructed, and the green fluorescence could be observed under the microscope after successful transfection of the plasmid. (g) The hnRNPG expression was upregulated, and the FOX2 expression was downregulated in IAH1 overexpressing cell lines. (h) Upregulation of IAH1 was found in cell lines overexpressing hnRNPG. (i) Downregulation of IAH1 was found in cell lines overexpressing FOX2. All data were representative of at least three independent experiments (n = 3; error bar, SD). Supplementary Figure 2: the effect of hnRNP G and FOX2 on the biological behavior of ESCA cell lines in vitro. (a) Invasion ability was measured in EC-109, and KYSE-150 transfected with another FOX2 and hnRNP G sequences by wound healing test. ∗P < 0.05 vs. cells transfected with control siRNA. (e) The bar graphs represent the mRNA expression level of EMT-related genes in EC-109 and KYSE-150 cell lines after transfected with another FOX2 and hnRNP G sequences. ∗P < 0.05, ∗∗P < 0.01, ∗∗∗P < 0.001. All data were representative of at least three independent experiments (n = 3; error bar, SD). [file 3043737.f1.docx]
